# The Evaluation of Degree of Monomer Conversion, Biaxial Flexural Strength, and Surface Mineral Precipitation of Orthodontic Adhesive Containing Sr-Bioactive Glass Nanoparticles, Calcium Phosphate, and Andrographolide

**DOI:** 10.3390/ma18102278

**Published:** 2025-05-14

**Authors:** Wirinrat Chaichana, Supachai Chanachai, Kanlaya Insee, Sutiwa Benjakul, Parichart Naruphontjirakul, Piyaphong Panpisut, Woranuch Chetpakdeechit

**Affiliations:** 1 Division of Orthodontics, Faculty of Dentistry, Thammasat University, Pathum Thani 12120, Thailand; 2 Biological Engineering Program, Faculty of Engineering, King Mongkut’s University of Technology Thonburi, Bangkok 10140, Thailand; 3 Division of Restorative Dentistry, Faculty of Dentistry, Thammasat University, Pathum Thani 12120, Thailand; 4 Thammasat University Research Unit in Dental and Bone Substitute Biomaterials, Thammasat University, Pathum Thani 12120, Thailand

**Keywords:** orthodontic adhesive, bioactive glass, calcium phosphate, polymerization, flexural strength, monomer conversion, surface mineral precipitation

## Abstract

This study examined the degree of monomer conversion (DC) and mechanical properties of experimental orthodontic adhesives containing monocalcium phosphate monohydrate (MCPM), Sr-bioactive glass (Sr-BAG) nanoparticles, and andrographolide. Experimental adhesives were prepared with a 4:1 powder-to-liquid ratio, containing methacrylate monomers with varying formulations of glass fillers and additives. DC was measured using ATR-FTIR (n = 5) with and without bracket placement under two curing protocols: conventional LED (1200 mW/cm^2^, 20 s) and high-intensity LED (3200 mW/cm^2^, 3 s). The biaxial flexural strength and modulus were tested after 4-week water immersion (n = 8). Transbond XT was used as the commercial comparison. Transbond XT exhibited higher DC (33–38%) than the experimental materials. Conventional LED curing produced higher DC than high-intensity LED, while bracket placement reduced DC by approximately 10% in the experimental materials but minimally affected Transbond XT. Transbond XT demonstrated a superior biaxial flexural strength (188 MPa) compared to the experimental adhesives (106–166 MPa, *p* < 0.05). However, the experimental formulations with low additive concentrations showed a comparable biaxial flexural modulus (5.0–5.5 GPa) to Transbond XT (5.6 GPa) (*p* > 0.05). Although the experimental adhesives exhibited lower DC and strength than the commercial product, their values still met the ISO standards, suggesting their potential clinical viability despite their modified compositions.

## 1. Introduction

White spot lesions are a common complication in 25–30% of patients treated with fixed orthodontic appliances [1]. If left untreated, these active demineralized lesions could lead to more severe cavitated lesions and interrupt the orthodontic treatment [2,3]. It was estimated that the population level direct costs of caries from 12–65 years of age could be varied between USD 10.2 and 36.2 billion [4]. Topical fluoride or fluoride-containing bonding systems appeared beneficial for prevention and treatment strategies [5]. Orthodontic adhesives, primarily resin-based composites, have been developed by enhancing their antibacterial and remineralization properties. Regarding the antibacterial properties, chlorhexidine has been extensively studied, but allergic reactions remain a major concern [6,7,8]. The incidence of allergic reaction to chlorhexidine mouthwash was unknown, but the prevalence of perioperative anaphylaxis was reported to be 0.05–2% [9].

Various approaches have been explored through the incorporation of ion-releasing components such as fluoride compounds, bioactive glass, and calcium phosphate nanoparticles. The addition of reactive fillers that promote the release of calcium or phosphate ions was expected to promote the remineralizing effect [10]. Monocalcium phosphate monohydrate (MCPM) has shown promising results in promoting hydroxyapatite formation; however, higher concentrations (10–40 wt%) may compromise the mechanical properties [10,11,12]. A previous study that prepared orthodontic adhesives revealed that the addition of MCPM, Sr-bioactive glass nanoparticles, and andrographolide reduced the degree of monomer conversion (DC) from 62% (control) to 47% in the experimental formulations [13]. The biaxial flexural strength decreased from 193 MPa (control) to 119 MPa at the highest additive content. The inadequate polymerization of resin-based materials may increase the risk of releasing unreacted monomers [14]. These monomers have the potential to exhibit genotoxicity, such as deleting DNA sequences in mammalian cells [15]. Additionally, they may cause caspase activation and increased reactive oxygen species (ROS) production, which can damage DNA, proteins, and lipids [15]. While the physical properties were decreased, the release of calcium, phosphorus, and strontium was observed. The increased concentration of MCPM and Sr-bioactive glass (Sr-BAG) nanoparticles also led to an 18% reduction in the growth of planktonic *S. mutans* [13].

The development of high-intensity LED units, such as VALO Grand (light intensity of 3200 mW/cm^2^, Ultradent Products Inc., South Jordan, UT, USA), was expected to provide similar polymerization compared with conventional LED curing [16]. This optimization and shortening of the curing protocols (3 s) become crucial for clinical efficiency. The variation in light intensity, exposure time, and wavelength could also significantly affect the orthodontic adhesive’s polymerization and mechanical strength [17]. It was demonstrated that the use of second-generation LED tended to result in a lower DC [17]. A sufficient curing time may be required to ensure that high polymerization and strength can be achieved [18]. The third-generation LED (polywave LED, ~1400 mW/cm^2^), which allows for shorter curing times, still achieved a higher level of polymerization and mechanical properties for resin composites compared to the second-generation LED (monowave LED, ~1200 mW/cm^2^) [18]. Additionally, it was revealed that the presence of metal brackets could reduce the polymerization of orthodontic adhesives compared to ceramic brackets or no brackets at all [6].

The objectives of this study were to investigate the effects of different curing protocols (conventional LED versus high-intensity LED) on the degree of monomer conversion (DC) in experimental orthodontic adhesives containing Sr-BAG nanoparticles, MCPM, and andrographolide. Furthermore, the study aimed to assess the impact of orthodontic bracket placement and curing protocol on DC, examine the biaxial flexural strength (BFS) and modulus (BFM) of the experimental materials following water aging, and analyze the fracture surface characteristics utilizing SEM-EDX analysis. The null hypotheses were as follows: (1) there is no significant difference in DC between conventional LED and high-intensity LED curing protocols; (2) the presence of an orthodontic bracket does not significantly affect the DC of the materials; (3) there is no significant difference in DC between experimental and commercial adhesives; and (4) there is no significant difference in BFS and BFM between experimental and commercial adhesives after water aging for 4 weeks.

## 2. Materials and Methods

### 2.1. Material Preparation

**Figure 1:**
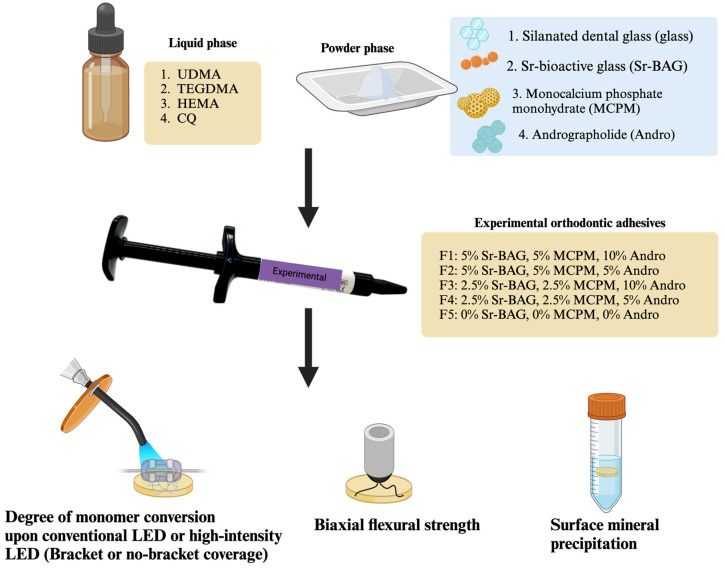
The experimental setup of the current study. Created in BioRender. Panpisut, P. (2025) https://BioRender.com/q26d152.

The experimental orthodontic adhesives were prepared using a powder-to-liquid ratio of 4:1 by weight (Figure 1). The liquid phase consisted of light-curable methacrylate monomers: 70 wt% urethane dimethacrylate (UDMA, Sigma-Aldrich, St. Louis, MO, USA), 26 wt% triethyleneglycol dimethacrylate (TEGDMA, Sigma-Aldrich, St. Louis, MO, USA), and 3 wt% 2-hydroxyethyl methacrylate (HEMA, Sigma-Aldrich, St. Louis, MO, USA). Additionally, 1 wt% camphorquinone (CQ, Sigma-Aldrich, St. Louis, MO, USA) was added as a photoinitiator.

The powder phase contained varying concentrations of components: silanized boroaluminosilicate glass (particle diameter ~7 μm and ~0.7 μm, Esstech Inc., Essington, PA, USA), monocalcium phosphate monohydrate (MCPM, particle diameter ~10 μm, Himed, Old Bethpage, NY, USA), Sr-bioactive glass nanoparticles (Sr-BAG nanoparticles, particle diameter ~200 nm, King Mongkut’s University of Technology Thonburi, Bangkok, Thailand), and andrographolide (Nanjing NutriHerb BioTech, Nanjing, China). Five experimental formulations (F1–F5) were prepared with varying concentrations of these components (Table 1).

The preparation of Sr-doped bioactive glass was reported in the previous study [19]. This composition was selected to optimize the ion release and bioactivity [19]. Spherical strontium–bioactive glass (Sr-BAG) nanoparticles with an approximate diameter of 200 nm were prepared using a sol–gel technique. The synthesis started with the formation of silica nanoparticles (SiO_2_-NPs) as a precursor material. The process involved combining 0.32 M ammonium hydroxide, 6 M Milli-Q water, and 14 M ethanol (99.5%) in a 500 mL Erlenmeyer flask with continuous stirring at 500 rpm for 10 min. Then, 0.28 M tetraethyl orthosilicate (TEOS, Sigma-Aldrich, St. Louis, MO, USA) was added dropwise to the mixture. The solution continued being stirred for 10 h to ensure complete hydrolysis and polycondensation reactions. The resulting SiO_2_-NPs were then collected and modified by incorporating 0.09 M calcium nitrate tetrahydrate (99%, Sigma-Aldrich, St. Louis, MO, USA) and 0.27 M strontium nitrate (99%, Merck, Darmstadt, Germany). The modified particles underwent calcination at 680 °C for 3 h, with a controlled heating rate of 3 °C/min. After calcination, the particles were then cleaned with ethanol.

The powder and liquid phases were hand mixed on a mixing pad using a plastic spatula for 30 s until a homogeneous consistency was achieved. The mixed adhesives were then loaded into 1 mL composite syringes (MIXPAC, medmix Switzerland AG, Haag, Switzerland) for ease of application. Transbond XT (3M Unitek, Monrovia, CA, USA) was used as a commercial control material (Table 2).

### 2.2. Degree of Monomer Conversion

The degree of monomer conversion (DC) of the materials (n = 5) was determined using attenuated total reflection Fourier transform infrared spectroscopy (ATR-FTIR, Nicolet iS5, Thermo Fisher Scientific, Waltham, MA, USA). Each uncured adhesive was placed in a metal circlip (1 mm thickness, 10 mm diameter) on the diamond of ATR. The materials were tested under two variables: with/without orthodontic bracket placement and two different light-curing protocols. For specimens without bracket placement, the materials were covered with an acetate sheet. For specimens with bracket placement, premolar brackets (GEMINI MBT 0.022 Twin, 3M Unitek, Monrovia, CA, USA) were positioned on top of the uncured adhesive. The setup of the experimental groups is described in Table 3. FTIR spectra were recorded in the region of 700–4000 cm^−1^ from the bottom surface of each specimen before and immediately after light curing. The resolution was 4 cm^−1^, and the measurement was set at 8 repetitions. The degree of monomer conversion was calculated using the following equation [19].

(1) $$DC=\frac{100(∆A_{0}-∆A_{t})}{∆A_{0}}$$

In this equation, $∆A_{0}$ and $∆A_{t}\;$ represent the peak height of the C-O stretching vibration of the methacrylate group at 1320 cm^−1^ above the background level at 1335 cm^−1^, measured before curing and at the time *t* after initiating curing, respectively [20].

**Table 3:** Experimental setup for degree of monomer conversion testing.

Group	Bracket Presence	Light-Curing Protocol	Time (On Each Mesial and Distal Side)	Manufacturer of Light-Curing Unit
NB-CLED	No	Conventional LED SmartLite (1200 mW/cm^2^)	20 s	DENTSPLY Sirona, York, PA, USA
NB-HLED	No	High-intensity LED Valo Grand (3200 mW/cm^2^)	3 s	Ultradent Products Inc., South Jordan, UT, USA
B-CLED	Yes	Conventional LED SmartLite (1200 mW/cm^2^)	20 s	DENTSPLY Sirona, York, PA, USA
B-HLED	Yes	High-intensity LED Valo Grand (3200 mW/cm^2^)	3 s	Ultradent Products Inc., South Jordan, UT, USA

### 2.3. Biaxial Flexural Strength and the Modulus of Elasticity

Disc specimens (n = 8) were prepared for biaxial flexural strength (BFS) and modulus (BFM) testing. The adhesive materials were placed in metal circlips (10 mm diameter, 1 mm thickness) between acetate sheets and glass slides. The specimens were light cured using the conventional LED protocol (SmartLite, Boca Raton, FL, USA, 1200 mW/cm^2^, 20 s) on both top and bottom surfaces to ensure the optimal polymerization. After curing, the specimens were removed from the molds and stored in deionized water at 37 °C for 4 weeks.

The biaxial flexural strength test was performed using a ball-on-ring testing jig mounted on a universal testing machine (AGSX, Shimadzu, Kyoto, Japan). Each specimen was positioned centrally on a support ring (8 mm diameter), and load was applied using a 500 N load cell with a crosshead speed of 1 mm/min until specimen failure. The maximum load at failure was recorded. Then, the biaxial flexural strength (BFS, Pa) and biaxial flexural modulus (Pa) were calculated using the following equations [19].

(2) $$BFS=\frac{F}{d^{2}}\left\{\left(1+v\right)\left[0.485ln\left(\frac{r}{d}\right)+0.52\right]+0.48\right\}$$

(3) $$BFM=\left(\frac{\Delta H}{\Delta W_{c}}\right)\times \left(\frac{\beta_{c}d^{2}}{q^{3}}\right)$$

Here, F is the failure load (N), d is the thickness of the disc specimens (m), r is the radius of the circular support of the ball-on-ring testing jig (m), and v represents Poisson’s ratio (0.3) [21]. $\frac{\Delta H}{\Delta W_{c}}$ is the rate of load change relative to the central deflection or gradient of force versus the displacement curve (N/m). $\beta_{c}$ and q are the center deflection function (0.5024) and the ratio of the support radius to the specimen radius, respectively.

After testing, representative fractured specimens were selected for examination of the fracture surfaces using scanning electron microscopy (SEM, JSM 7800F, JEOL Ltd., Tokyo, Japan) to determine the mixing quality. The specimens were sputter coated with gold (Q150R, Quorum Technologies, East Sussex, UK) before SEM analysis. The test was performed at 10 kV.

### 2.4. Surface Mineral Precipitation

Disc specimens with the dimension of 10 mm in diameter and 1 mm in thickness were prepared. This test was a qualitative analysis for surface mineral precipitation (n = 1). The disc specimen was placed in 5 mL of simulated body fluid (SBF) prepared according to ISO BS ISO 23317:2014 Implants for surgery: in vitro evaluation for the apatite-forming ability of implant materials [19]. The disc was immersed for 4 weeks in an incubator with a controlled temperature (37 °C). Then, the disc was removed and sputter coated with gold. The mineral precipitation on the surface was analyzed using SEM equipped with EDX (EDX, X-sight 6650 detector, Oxford Instruments, Abingdon, UK). The test was performed using 10 kV.

### 2.5. Statistical Analysis

All the numerical data were analyzed using GraphPad Prism version 9.3 for macOS (GraphPad Software, San Diego, CA, USA). The normality of data distribution was evaluated using the Shapiro–Wilk test. The sample size for each test was calculated using G*Power 3.1 software (University of Dusseldorf, Germany) [22] based on the previous published studies and pilot data, indicating power > 0.95 at α = 0.05. The degree of monomer conversion and biaxial flexural strength results were compared among the groups using one-way ANOVA followed by Tukey’s multiple comparisons test to examine the differences between the curing protocols and bracket placement conditions. The effects of the curing protocols, type of orthodontic adhesives, and bracket coverage on DC were analyzed using a generalized linear model.

## 3. Results

### 3.1. Degree of Monomer Conversion

**Figure 2:**
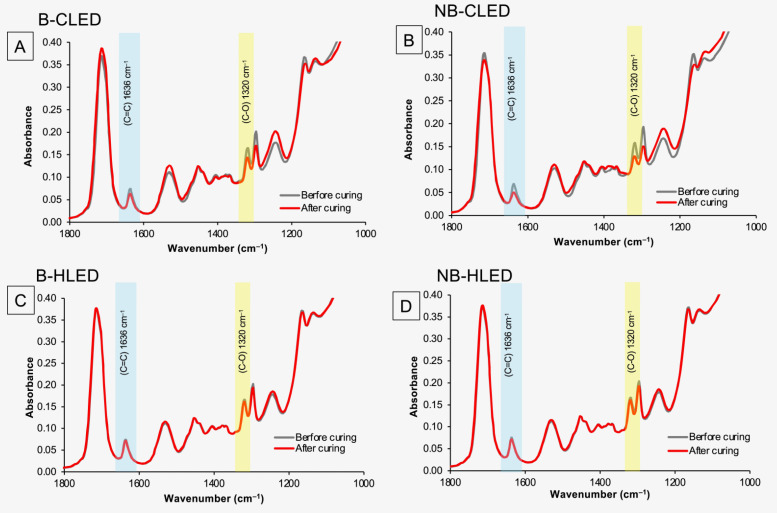
(**A**) FTIR spectra before and after curing with a conventional LED with bracket coverage of F1. (**B**) FTIR spectra before and after curing with a conventional LED without bracket coverage of F1. (**C**) FTIR spectra before and after curing with a high-intensity LED with bracket coverage of F1. (**D**) FTIR spectra before and after curing with a high-intensity LED without bracket coverage of F1.

The reduction in FTIR peaks that represent methacrylate groups such as C-O, 1320 cm^−1^, or C=C, 1636 cm^−1^ could be observed after light curing in all materials (Figure 2). However, the reduction in the peaks for experimental materials was greater when the materials were cured with a conventional light-curing protocol (CLED).

**Figure 3:**
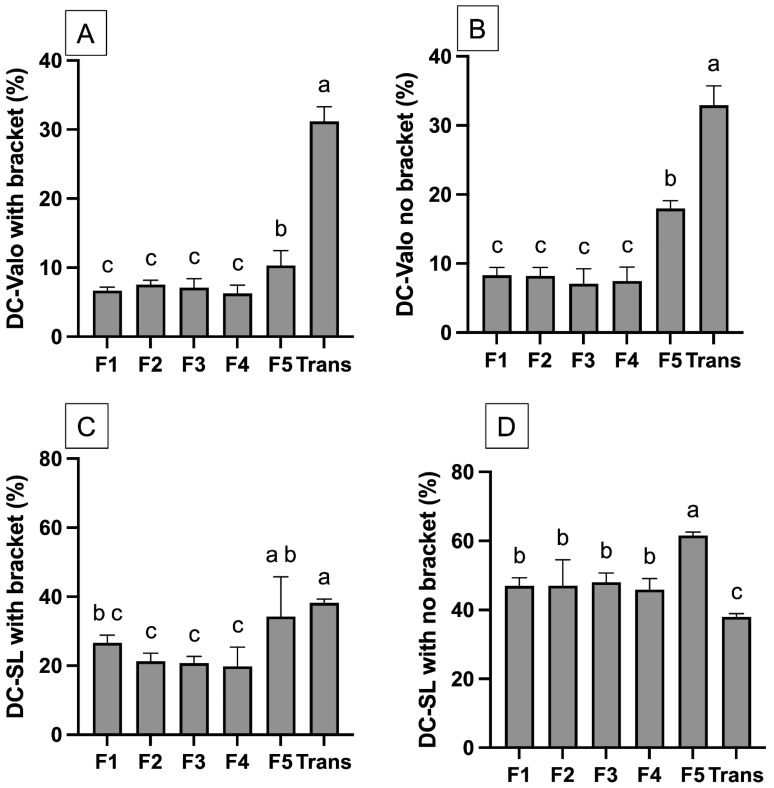
(**A**) The DC of materials curing with a high-intensity LED with bracket coverage. (**B**) The DC of materials curing with a high-intensity LED without bracket coverage. (**C**) The DC of materials curing with a conventional LED with bracket coverage. (**D**) The DC of materials curing with conventional LED without bracket coverage. The same letters indicate *p* > 0.05. Error bars are 95%CI (n = 5).

For the measurement with bracket coverage (Figure 2C and Figure 3A) the highest value was observed with Trans. The DC of Trans (31.2 ± 1.7%) for HLED was significantly higher than that of the other experimental materials (*p* < 0.05). The lowest DC was recorded for F1 (6.7 ± 0.4%), which was comparable to F2 (7.5 ± 0.5%, *p* = 0.8451), F3 (7.1 ± 1.1%, *p* = 0.9912), and F4 (6.3 ± 0.9%, *p* = 0.9953). The DC of F1–F4 was also significantly lower than F5 (10.3 ± 1.7%) (*p* < 0.05). All the materials exhibited a significantly higher DC when cured with CLED (*p* < 0.05). The DC of Trans (38.2 ± 9.3%) was comparable to that of F5 (34.2 ± 9.3%) (*p* = 0.6997). The DC value of F1 (26.7 ± 1.8%) was also comparable to F2 (21.3 ± 1.9%, *p* = 0.4172), F3 (20.8 ± 1.5%, *p* = 0.3163), and F4 (19.8 ± 4.5%, *p* = 0.4172).

For the measurement without bracket coverage (Figure 2D and Figure 3B), the observed DC of materials cured with CLED was higher than that observed with HLED. For CLED, the highest DC was detected with F5 (61.6 ± 0.8%), which was significantly higher than that of other materials (*p* < 0.05). The DC of Trans (38.0 ± 0.8%) was significantly lower than F1 (46.9 ± 1.9%, *p* < 0.01), F2 (46.9 ± 1.9%, *p* < 0.01), F3 (46.9 ± 1.9%, *p* < 0.01), and F4 (45.9 ± 2.6%, *p* < 0.01). However, for HLED, the DC of Trans (33.0 ± 2.3 %) was significantly higher than that of the other materials (*p* < 0.05). F5 (18.0 ± 2.3%) also demonstrated the highest DC compared to F1 to F4 (~7–8%).

The results from the generalized linear model indicate that the light-curing protocol (*p* < 0.01), bracket coverage (*p* < 0.01), type of orthodontic adhesive (*p* < 0.01), and all the interaction effects (*p* < 0.01) significantly affected the DC of the tested materials.

### 3.2. Biaxial Flexural Strength (BFS) and the Biaxial Flexural Modulus (BFM)

**Figure 4:**
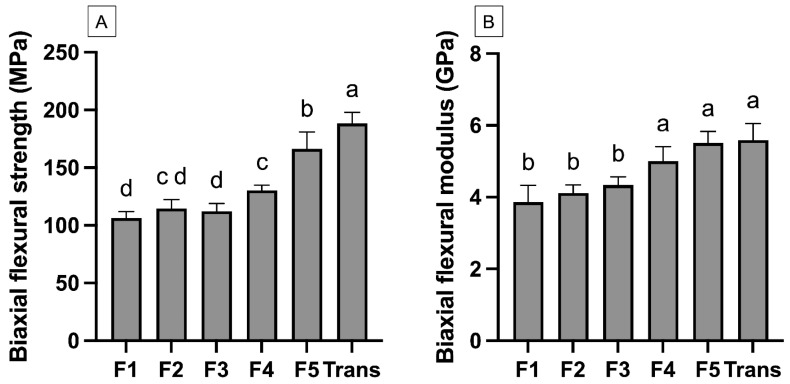
(**A**) Biaxial flexural strength after immersion in water for 4 weeks. (**B**) biaxial flexural modulus of materials after immersion in water for 4 weeks. The same letter indicates *p* > 0.05. The same letters indicate *p* > 0.05. Error bars are 95%CI (n = 8).

**Figure 5:**
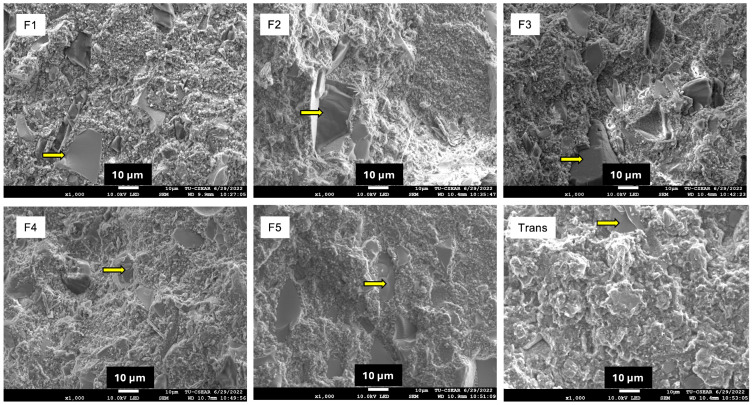
SEM image of tested specimens. The arrows indicate glass fillers embedded in the resin matrix.

The highest BFS was detected with Trans (189 ± 11 MPa) (Figure 4), while the lowest value was obtained from F1 (106 ± 7 MPa) (Figure 3A). Trans exhibited significantly higher BFS than all the experimental materials (*p* < 0.05). The BFS of F1 (106 ± 7 MPa) was comparable to F2 (115 ± 9 MPa) (*p* = 0.6067) and F3 (112 ± 8 MPa) (*p* = 0.8749). Similarly, the highest BFM was observed with Trans (5.6 ± 0.5 GPa) (Figure 3B), but this value was comparable to F5 (5.5 ± 0.3 GPa) (*p* = 0.99) and F4 (5.0 ± 0.4 GPa) (*p* = 0.09). The fracture surface (Figure 4) of the tested materials showed fillers embedded in the resin matrix. Smaller and more homogeneously mixed fillers were detected in Trans (Figure 5).

### 3.3. Surface Mineral Precipitation

**Figure 6:**
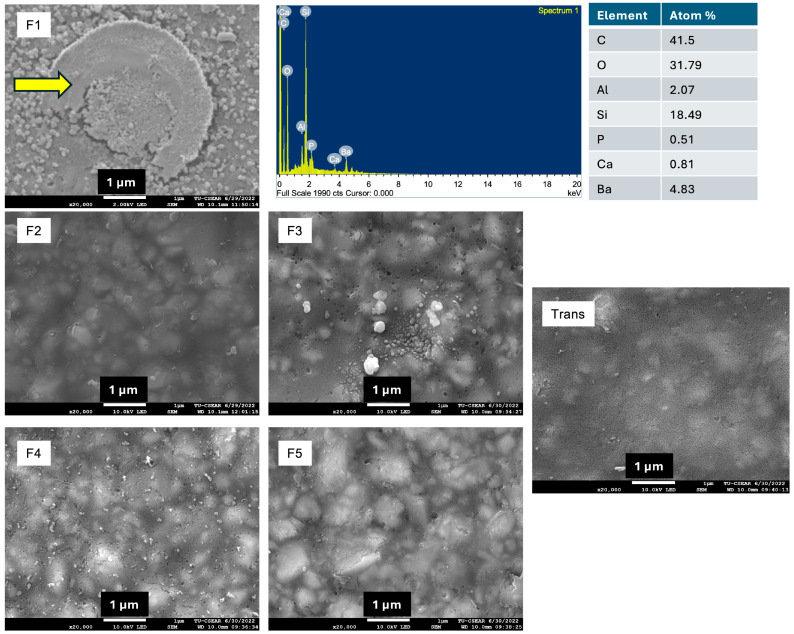
The surface of materials after immersion in simulated body fluid (SBF) for 4 weeks. Calcium phosphate precipitation (arrow) was detected only on the surface of F1.

The SEM image showed that only F1 exhibited the precipitation of minerals on the surface (Figure 6). The elemental analysis indicated that the precipitate could be calcium phosphate.

## 4. Discussion

This study investigated the effect of different light-curing protocols (conventional LED-curing protocol versus a high-intensity LED protocol) on the degree of monomer conversion of the experimental orthodontic adhesives. The results showed that different curing protocols and bracket placement influenced the polymerization of the experimental materials. Additionally, the strength between the experimental materials and commercial materials was significantly different. Hence, all null hypotheses were rejected.

The use of a high-intensity light-curing protocol is beneficial for placing orthodontic brackets as it can significantly shorten the chair time [23]. The current study indicated that the commercial material (Transbond XT) was minimally affected by different light-curing protocols and bracket placement techniques. This could be attributed to Transbond XT’s higher translucency compared to the experimental orthodontic adhesives, which facilitates better light penetration through the material. These findings align with the previous study, which demonstrated that curing with either the conventional LED protocol (1200 mW/cm^2^ for 20 s) or high-intensity LED protocol (3200 mW/cm^2^ for 6 s) resulted in comparable bond failure rates (1.18% for both protocols) [23].

The experimental orthodontic adhesives exhibited much lower DC when applying a high-intensity light-curing unit for 3 s. The DC of the experimental adhesives increased substantially when the curing time was extended to 20 s with conventional LED, suggesting that these materials require sufficient polymerization time to achieve the optimal properties. The increase in DC observed when using conventional LED was expected to help reduce the risk of toxic monomer release from the adhesives [14]. This is particularly important as inadequate polymerization and subsequent monomer leaching may enhance the biofilm accumulation, potentially increasing the risk of white spot lesions around orthodontic brackets [24]. This extended curing duration may likely promote the complete polymerization or alteration of monomers, which may increase their translucency or help reduce the light-scattering effect within the material [25]. This was consistent with a previous study that compared the DC of resin composites when cured using a conventional LED light-curing protocol (1180 mW/cm^2^ for 20 s) and a high-intensity LED (3150 mW/cm^2^ for 3 s) [26]. The study indicated that lower irradiation with a longer exposure time is recommended to achieve a high DC [26].

The explanation for the low DC of the experimental materials under high-intensity LED light may be their lower viscosity compared to commercial materials. The apparent viscosity of the experimental materials after mixing was slightly lower than that of the commercial product. However, an actual viscosity analysis should be confirmed using a rheometer in future studies. A previous study indicated that composites with lower viscosity exhibited a significant reduction in surface microhardness by 11–48% when cured with high-intensity LED light, as opposed to conventional light curing methods [27]. It was suggested that high-intensity LED light might cause a rapid and substantial production of free radicals [27]. The high concentration of free radicals in a low-viscosity polymerizing mixture increases the likelihood of bimolecular termination [28], where free radicals combine with each other instead of forming long, strong polymer chains. This premature termination of the polymerization process may result in a low DC and a less well-formed network of polymer chains.

The biaxial flexural strength of the experimental orthodontic adhesives after immersion in water for 4 weeks decreased upon the addition of reactive components. The minimum clinical requirement of flexural strength for orthodontic adhesives has not been established. The lowest observed result was with F1 (106 MPa), which is still higher than the 50 MPa required for a type 2 resin composite used as a luting agent, according to BS EN ISO 4049:2019, Dentistry—Polymer-based restorative materials [29]. The additives reduced the strength of the materials, as was expected. This was possibly due to the lack of silanization of the reactive components. The additives reduced the flexural strength by approximately by 50%. This may be correlated with the result from the previous study. It was demonstrated that the use of the silanization of fillers could increase the strength from 71.1 MPa to 112 MPa (~57%) due to the enhancement of the chemical bond between the fillers and resin matrix [30]. However, the additives were designed not to be silanized to ensure that their bioactive properties, such as ion-releasing actions, are not affected. It was suggested that the silanization of reactive fillers may reduce the ion release due to the presence of strong chemical bonds with the resin matrix, which hinders the dissolution of the fillers [31]. However, some studies indicated that the silanization of reactive fillers could enhance the ion release and remineralization [32,33]. This enhancement may be attributed to the process of slightly dissolving the surface of the particles [32], thereby increasing the release of ions from the fillers.

The lower strength of the adhesive could be due to the low DC, which may reduce the rigidity of the polymer network, which may be susceptible to the water plasticization of the hydrophilic components [34]. The previous study showed that the lowest shear bond strength to enamel at 24 h for the experimental materials was approximately 20 MPa [19], which is higher than the clinically acceptable values of around 5.9–7.8 MPa [35]. Future tests should also assess the long-term shear bond strength and the effect on the bonded enamel [36] of the materials to ensure their clinical acceptability.

The release of ions from the experimental materials was expected to promote mineral precipitation, which could potentially help enhance the enamel remineralization [37]. Only F1 demonstrated detectable surface mineral precipitation, which could be due to the high level of additives used. The level of mineral precipitation was thin, which could be due to the slow and low-level release of calcium and phosphate ions from MCPM and bioactive glass [19]. The previous study measured the release of Ca and P from the specimens in water over 4 weeks. High levels of Ca and P release, which corresponded with high water absorption and solubility, were observed in F1 and F2 [19]. However, precipitation was primarily detected in F1, though the detection area was limited. This discrepancy between the ion release and precipitation in the current study may be attributable to the differences in the surface characteristics between the specimens. It was indicated that the surface topography and irregularities affected the surface apatite formation on biomaterials [38]. Future work should, therefore, include an examination of the surface roughness parameters (Ra) to systematically investigate the relationship between the surface topography and apatite formation. The release kinetics of elements in the long term should also be determined in future work. Additionally, the pH of SBF was set at ~7.5, which is higher than the critical pH of enamel (~5.5) [39]. The previous study demonstrated the placement of an orthodontic adhesive disc containing bioactive glass in the demineralized and remineralized buffer for up to 180 days [40]. The same study reported that the surface apatite formation increased over time, particularly in acidic conditions. This character was expected to promote preventive effects for demineralized enamel. Future work should employ a longer immersion period using the demineralization/remineralization cycles.

The use of hydrophilic fillers and the hand-mixing process for experimental orthodontic adhesive raises concerns about the potential voids and air bubbles in the bulk of the materials. Hence, the fracture surface of the specimens was investigated. The SEM images of the fracture surface showed minimal voids in the specimens. This observation suggests that reactive fillers may slowly dissolve, potentially reducing the formation of voids on the surface. Despite the minimal voids detected, hand-mixing methods may not provide consistent quality control over the mixed paste. Therefore, the use of a vacuum planar mixing machine might be required in future studies. The presence of voids in the materials can also be quantified in future work by using micro-CT analysis [41].

The results in the current study may not yet be sufficient to identify a suitable candidate formulation for further clinical trials. Andrographolide and Sr-bioactive glass were expected to control the formation of biofilms [42,43]. Future studies should investigate the ability of the experimental orthodontic adhesives to inhibit the formation of biofilms [44]. According to the result from the current study, F1, which demonstrated surface mineral precipitation, could be considered suitable for further analysis. The use of a conventional light-curing protocol for the experimental adhesives may be recommended for reducing the risk of suboptimal polymerization. The results from the current study indicate that the use of bracket coverage led to the reduction in polymerization of the underlying orthodontic adhesives. It may be recommended that the assessment of DC for orthodontic adhesives should be performed under brackets to mimic the clinical condition of the material.

## 5. Conclusions

The experimental orthodontic adhesives demonstrated a higher degree of monomer conversion when cured with a conventional LED light-curing protocol (1200 mW/cm^2^ for 20 s) compared to using a high-intensity light-curing protocol (3200 mW/cm^2^ for 3 s). This may indicate that the use of the conventional LED light-curing protocol is recommended with the experimental materials. The placement of orthodontic brackets also detrimentally reduced the polymerization of the materials. The strength of the experimental materials was lower than that of the commercial materials; however, these values remained within the acceptable range required by the ISO standard. Future work should examine the effects of curing protocols on the bond strength to enamel, enamel remineralization, and biofilm modulation of the materials.

## Figures and Tables

**Table 1:** Composition of the experimental orthodontic adhesive. All formulations contain the same liquid phase.

Composition (wt%)	F1	F2	F3	F4	F5
Boroaluminosilicate glass (diameter of 0.7 and 7 μm)	80	85	85	90	100
Sr-bioactive glass (Sr-BAG) nanoparticles	5	5	2.5	2.5	0
Monocalcium phosphate monohydrate (MCPM)	5	5	2.5	2.5	0
Andrographolide	10	5	10	5	0

**Table 2:** Composition of the commercial orthodontic adhesive.

Material	Composition (wt%)	Supplier
Transbond XT	45–55 wt% Bisphenol A Diglycedyl Ether Dimethacrylate (Bis-GMA), 45–55 wt% Triethylene glycol dimethacrylate, 4-(dimethylamino)-benzeneethanol	3M Unitek, Monrovia, CA, USA

## Data Availability

The raw data supporting the conclusions of this article will be made available by the authors on request.

## Abbreviations

The following abbreviations are used in this manuscript:

ATR-FTIR	Attenuated total reflection Fourier transform infrared spectroscopy
B	Bracket coverage
BAG	Bioactive glass
BFM	Biaxial flexural modulus
BFS	Biaxial flexural strength
CLED	Conventional LED curing
CQ	camphorquinone
DC	Degree of monomer conversion
EDX	Energy dispersive X-ray
HEMA	2-hydroxyethyl methacrylate
HLED	High-intensity LED curing
ISO	International organization for standardization
LED	Light-emitting diode
MCPM	Monocalcium phosphate monohydrate
NB	No bracket coverage
SEM	Scanning electron microscope
TEGDMA	triethyleneglycol dimethacrylate
Trans	Transbond XT
UDMA	urethane dimethacrylate
